# Comparative Study of Corrosion Inhibition Properties of Q345 Steel by Chitosan MOF and Chitosan Schiff Base

**DOI:** 10.3390/ma18133031

**Published:** 2025-06-26

**Authors:** Lizhen Huang, Jingwen Liu, Li Wan, Bojie Li, Xianwei Wang, Silin Kang, Lei Zhu

**Affiliations:** 1 School of Civil Engineering, Hubei Engineering Research Center for Cement-Based Ultra-High-Performance Concrete and Prefabricated Building Technology, Hubei Engineering Research Center for Key Technologies in Modern Paper and Sanitary Products Manufacturing, Hubei Engineering University, Xiaogan 432000, China; hlizhen@hbeu.edu.cn (L.H.);; 2 School of Physics and Electronic Information Engineering, Ningxia Normal University, Guyuan 756099, China

**Keywords:** Q345 steel, chitosan–copper metal–organic framework (CS@Cu MOF), chitosan–Schiff base–copper functional material (Schiff–CS@Cu), chloride–acid corrosion, corrosion inhibition efficiency

## Abstract

This study synthesized two eco-friendly inhibitors—a chitosan–copper metal–organic framework (CS@Cu MOF) and chitosan–Schiff base–Cu complex (Schiff–CS@Cu)—for Q345 steel protection in 3.5% NaCl/1M HCl. Electrochemical and weight loss analyses demonstrated exceptional corrosion inhibition: untreated specimens showed a 25.889 g/(m^2^·h) corrosion rate, while 100 mg/L of CS@Cu MOF and Schiff–CS@Cu reduced rates to 2.50 g/(m^2^·h) (90.34% efficiency) and 1.67 g/(m^2^·h) (93.56%), respectively. Schiff–CS@Cu’s superiority stemmed from its pyridine–Cu^2+^ chelation forming a dense coordination barrier that impeded Cl^−^/H^+^ penetration, whereas CS@Cu MOF relied on physical adsorption and micro-galvanic interactions. Surface characterization revealed that Schiff–CS@Cu suppressed pitting nucleation through chemical coordination, contrasting with CS@Cu MOF’s porous film delaying uniform corrosion. Both inhibitors achieved optimal performance at 100 mg/L concentration. This work establishes a molecular design strategy for green inhibitors, combining metal–organic coordination chemistry with biopolymer modification, offering practical solutions for marine infrastructure and acid-processing equipment protection.

## 1. Introduction

With the rapid development of marine engineering and infrastructure construction, Q345 low-alloy high-strength steel has been widely used in bridges, offshore platforms, and other key structures due to its excellent mechanical properties; however, its corrosion in chloride-containing acidic environments poses a serious threat to structural durability [1,2,3]. Although traditional organic corrosion inhibitors can effectively inhibit corrosion, they have issues such as high toxicity and poor biodegradability, which are not consistent with the development needs of green chemistry [4,5,6].

As a natural cationic polysaccharide, chitosan has demonstrated great potential in the field of environmentally friendly corrosion inhibitors by virtue of its abundant hydroxyl/amino functional groups and metal coordination ability [7,8]. In detail, chitosan can inhibit corrosion by adsorbing on the metal surface to form a protective film [9,10,11]. Studies have revealed that chitosan and its derivatives have a good corrosion inhibition effect on carbon steel in acidic media [12,13,14,15]. For instance, water-soluble chitosan derivatives exhibited excellent corrosion inhibition properties on mild steel in 1 M HCl solution [12]. Another study also confirmed the potential of modified chitosan as an efficient corrosion inhibitor for carbon steel in acidic media [10,13]. However, its inherent defects such as poor solubility and insufficient adsorption stability have restricted practical engineering applications [16,17]. In recent years, metal–organic frameworks (MOFs) and Schiff base compounds have attracted much attention in the field of functional materials due to their unique porous structure, high specific surface area, and designability [18,19,20,21,22].

It has been revealed that MOF materials can synergistically enhance corrosion inhibition performance through physical adsorption and chemical bonding [19,20]. The application of MOFs in the field of corrosion inhibition can hinder the penetration of corrosive media by forming a dense protective layer on the metal surface through physical or chemical adsorption [23,24]. It was found that MOFs synthesized from different metal precursors have enhanced corrosion resistance for mild steel in acidic media [24]. The structural properties of MOFs enable them to effectively trap and segregate corrosive ions, which further enhances the corrosion inhibition effect [23]. For example, excellent impedance matching can be achieved through compositional modulation engineering, and rich heterogeneous interfaces can be constructed to realize long-term corrosion protection [25].

Schiff base compounds have been widely used as metal corrosion inhibitors because of their ease of synthesis, variety of molecular structures, and the presence of heteroatoms (i.e., N, O, S) [26,27,28,29,30]. They usually form coordination bonds between the lone pair of electrons in the molecule and the empty orbitals on the metal surface, adsorb on the metal surface, and form a monomolecular or multimolecular protective film, thus inhibiting the electrochemical reaction [26,27,28,31]. Phenolic Schiff bases showed effective corrosion inhibition properties on mild steel in acidic environments [26]. Thiophene-based chitosan Schiff bases also exhibited corrosion inhibition in 1 M HCl solution [15]. Meanwhile, the corrosion inhibition efficiency of Schiff base compounds is closely related to its molecular structure, substituent type and position, concentration and ambient temperature, etc. [29,32].

The introduction of a Schiff base structure into chitosan chain through a Schiff base reaction could endow chitosan with better metal coordination ability and adsorption properties [33,34,35]. Through combining chitosan or Schiff base-modified chitosan with metal ions or MOFs, it is expected to construct composite corrosion inhibitors with synergistic effects, integrating the environmental friendliness of biopolymers, the porous structure of MOFs, and the strong adsorption ability of Schiff base to realize more efficient and long-lasting corrosion inhibition effects [33,36,37,38].

In this paper, two types of corrosion inhibitors, a chitosan–copper metal–organic framework (CS@Cu MOF) and a chitosan–Schiff Base–copper functional material (chitosan–3-pyridinecarbaldehyde Schiff Base–CuSO_4_ complex, Schiff–CS@Cu), were innovatively designed and synthesized, and their corrosion inhibition behaviors were systematically investigated on Q345 steel in a mixture of 3.5% NaCl and 1 mol/L HCl. Through the weight loss method, corrosion morphology characterization, and corrosion inhibition efficiency calculation, the concentration–performance correlation law was revealed, and the differences in the mechanism of action of the two types of corrosion inhibitors were compared, which provided a theoretical basis and technical path for the development of highly efficient and environmentally friendly protective materials for steel structures.

## 2. Materials and Methods

### 2.1. Experimental Materials

#### 2.1.1. Steel Specimens

**Figure 1:**
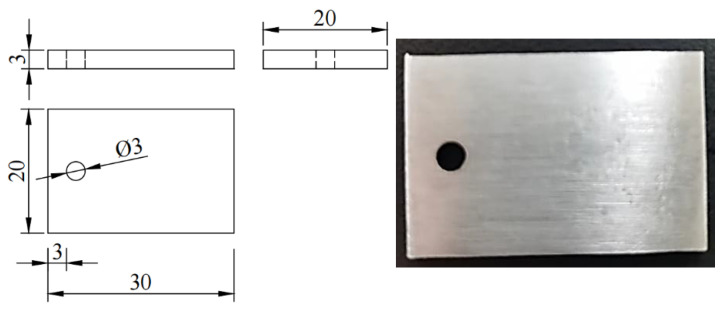
The images of steel specimen.

Q345 low-alloy high-strength steel (composition: C 0.18%, Mn 1.50%, Si 0.55%, P ≤ 0.025%, S ≤ 0.020%, Fe balance) was machined into rectangular plates (30 mm × 20 mm × 3 mm). Prior to testing, all specimens were sequentially polished with 400–2000 grit SiC abrasive paper to achieve a mirror finish (Ra ≤ 0.1 μm), degreased ultrasonically in acetone for 15 min, and dried under nitrogen flow. The specimen image is depicted in Figure 1.

#### 2.1.2. Corrosion Inhibitors

(a) Chitosan–copper MOF (CS@CU MOF): A chitosan–copper precursor solution was initially prepared by completely dissolving 2.0 g of chitosan powder in 50 mL of a 1 wt% acetic acid aqueous solution. This dissolution occurred under constant magnetic agitation at 400 rpm and 25 °C for 12 h. Subsequently, 5.7 g of Cu(NO_3_)_2_·3H_2_O was introduced into this homogeneous mixture, with stirring maintained at 400 rpm for an additional 2 h to achieve full complexation. The resulting solution underwent controlled gelation by careful dripping into a 3 M NaOH solution held at 25 °C. Following a 6 h reaction period, this process yielded spherical porous beads, which were then collected via vacuum filtration. A rigorous purification protocol ensued, involving rinsing with distilled water until neutral pH was attained, followed by sequential 20 min ultrasonication treatments in ethanol/water solutions with progressively increasing ethanol concentrations (10/90, 30/70, 50/50, 70/30, and 90/10 *v*/*v*), culminating in a final 20 min immersion in anhydrous ethanol to stabilize the pore structure. To induce MOF crystallization, the purified beads were immersed in a 0.16 g/L dimethylimidazole (DMIM) ethanol solution and reacted at 40 °C for 24 h under a nitrogen atmosphere. The final CS@Cu MOF material was obtained after vacuum drying at 60 °C for 12 h [39,40].
(b) Chitosan Schiff base (chitosan–3-pyridinecarbaldehyde Schiff base–CuSO_4_) complex (Schiff–CS@Cu): The preparation of chitosan–Schiff base–copper functional material (Schiff–CS@Cu) involves a two-step procedure. Initially, chitosan powder was dissolved in acetic acid solution to form a homogeneous system, which was then mixed with 3-pyridinecarboxaldehyde in anhydrous ethanol. The mixture underwent 12 h reflux condensation at 75 °C to form Schiff base linkages. The resultant Schiff–CS product was purified through sequential ethanol washing until colorless filtrate and deionized water rinsing to neutrality, followed by vacuum drying at 50 °C. Subsequently, the Schiff–CS material was immersed in saturated copper sulfate solution under 50 °C stirring for 6 h to achieve copper ion adsorption. After filtration and thorough washing to remove unbound ions, the final Schiff–CS@Cu composite was obtained through 12 h drying at 50 °C. This methodology integrates covalent modification through Schiff base formation with subsequent metal ion coordination, demonstrating the effective integration of organic–inorganic hybrid functionalities [41,42].

#### 2.1.3. Corrosive Medium

A simulated industrial marine environment was prepared by mixing 3.5 wt% NaCl (analytical grade) and 1 mol/L HCl (37% purity), yielding a solution with pH = 1.2 ± 0.1 (measured by pH meter, Mettler Toledo, Changzhou, China) [43].

### 2.2. Testing Methods

#### 2.2.1. Experimental Design

Six inhibitor concentrations (30, 50, 80, 100, 200, 300 mg/L) were tested for each inhibitor type, with triplicate specimens per group. Control groups (no inhibitor) were included for baseline comparison. Specimens were coded as M-30 to M-300 (CS@Cu MOF) and X-30 to X-300 (Schiff–CS@Cu), corresponding to their concentrations.

#### 2.2.2. Corrosion Testing

In this study, different concentrations of corrosion inhibitors were first added to the acidic corrosion medium (3.5 wt% NaCl (analytical grade) and 1 mol/L HCl (37% purity)) to form a mixed solution, and subsequently, the Q345 steel samples were immersed to a corrosion chamber configured with the above mixed solution and were subjected to a continuous corrosion process under the given conditions (25 ± 0.5 °C, 95 ± 2% RH) for 4 h. Post-corrosion, specimens were subjected to derusting per “Corrosion of metals and alloys: Removal of corrosion products from corroded specimens” (Chinese industry standard, GB/T 16545-2015) [44].

In detail, the specimen was firstly immersed in 10% HCl + 3.5 g hexamethylenetetramine solution (1 L) for 5 min to remove corrosion products. Then, it was ultrasonically cleaned in deionized water (10 min) and absolute ethanol (5 min). Finally, it was dried at 50 °C for 2 h and weighed (analytical balance, ±0.1 mg, Sartorius CPA225D, Changzhou, China).

#### 2.2.3. Data Analysis

Assuming that the specimen was uniformly corroded, the corrosion rate, *v* (g/(m^2^·h)) can be expressed as the weight loss of the metal after corrosion per unit of time and per unit of area, which was calculated as shown in Equation (1):

(1) $$v=\frac{m_{1}-m_{2}}{S\cdot t}$$

where *m*_1_ stands for the mass of the specimen before corrosion, g;

*m*_2_ is the mass of the specimen after corrosion, g;

*S* refers to the exposed area of the specimen, m^2^;

*t* denotes the time of exposure, h.

The corrosion inhibition rate, *η* (%), was calculated in Equation (2):

(2) $$\eta =\frac{\nu_{0}-\nu_{1}}{\nu_{0}}\times 100\%$$

where *v*_0_ and *v*_1_ denote corrosion rates of control and inhibited groups, respectively.

Statistical significance was assessed via one-way ANOVA (*p* < 0.05) using OriginPro 2018.

## 3. Results and Discussion

### 3.1. Corrosion Inhibition Effect

According to the data presented in Table 1, the corrosion rate of the blank control group (B-0) reached 25.889 g/(m^2^·h), indicating a severe degradation of the metal substrate in the corrosive medium. In contrast, experimental groups incorporating corrosion inhibitors at varying concentrations demonstrated remarkable improvements. The CS@Cu MOF group exhibited a corrosion rate which reduced to 2.000~3.167 g/(m^2^·h), representing a substantial reduction of 87.74~92.27%, while the Schiff–CS@Cu group showed a further decrease to 1.333~2.833 g/(m^2^·h), corresponding to an impressive inhibition efficiency of 89.06~94.85%. This concentration-dependent inhibitory effect confirms that both organic–inorganic composite corrosion inhibitors effectively interrupted the corrosion process through synergistic mechanisms. Specifically, the CS@Cu MOF likely utilizes the porous structure of the metal–organic framework to adsorb corrosive ions and establish a physical barrier layer, whereas the Schiff base groups in Schiff–CS@Cu form a chemisorbed protective layer on the metal surface via coordination interactions. These dual mechanisms collaboratively suppress both anodic metal dissolution and cathodic oxygen reduction reactions.

**Figure 2:**
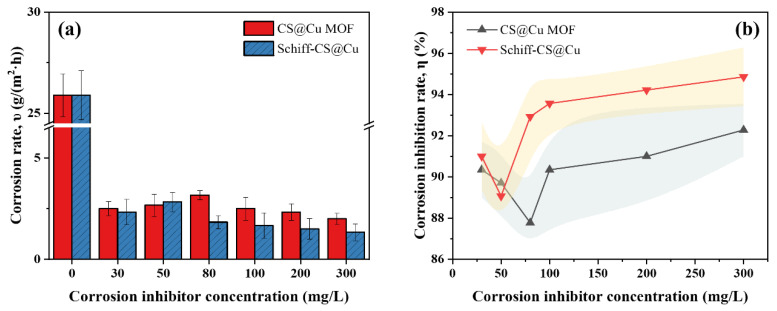
Corrosion rate and corrosion inhibition rate of samples with different corrosion inhibitors: (**a**) corrosion rate, (**b**) corrosion inhibition rate.

As illustrated in Figure 2a, the corrosion behavior of the CS@Cu MOF group exhibited a distinct non-monotonic concentration dependence. At 80 mg/L, the corrosion rate unexpectedly increased to 3.167 g/(m^2^·h), suggesting a critical threshold where insufficient inhibitor coverage or competitive adsorption between inhibitor molecules and corrosive species might compromise protection efficiency. However, at elevated concentrations (100–300 mg/L), the corrosion rates progressively decreased, reaching a minimum of 2.000 g/(m^2^·h) at 300 mg/L. This recovery implies that higher concentrations enable complete surface coverage through multilayer adsorption, effectively blocking active corrosion sites. In contrast, the Schiff–CS@Cu group demonstrated a linearly decreasing corrosion rate (slope *k* = −0.00167, R^2^ = 1) across the tested concentration range (100~300 mg/L), achieving optimal protection (1.333 g/(m^2^·h)) at 300 mg/L. This linear trend highlights its superior dose-responsive behavior, likely attributed to the Schiff base’s strong chemisorption capability that ensures uniform monolayer formation even at lower concentrations.

Furthermore, the corrosion inhibition efficiency (*η*, %) was quantitatively evaluated using Equation (2). As depicted in Figure 2b, both inhibitors showed concentration-dependent *η* enhancement. CS@Cu MOF exhibited fluctuating efficiency (peak *η* = 92.27% at 300 mg/L), aligning with its non-monotonic corrosion rate profile, while Schiff–CS@Cu displayed near-linear efficiency growth (*η* = 94.85% at 300 mg/L). The divergence in *η* trends underscores mechanistic differences: CS@Cu MOF’s porous framework may require critical concentration for optimal pore filling and ion trapping, whereas Schiff–CS@Cu’s planar molecular geometry facilitates rapid surface passivation through covalent bonding.

From Figure 2b, it can be found that CS@Cu MOF has the highest corrosion inhibition efficiency (*η* = 92.27%) at 300 mg/L, while Schiff–CS@Cu has a *η* of 94.85% at 300 mg/L, which is better than the former. Specifically, for the CS@Cu MOF group, the corrosion inhibition rate showed a “U-shape” change with the increase in concentration; the trough of the corrosion inhibition efficiency (*η* = 87.77%) occurred at 80 mg/L, which was mainly due to the fact that CS@Cu MOF physically adsorbed Cl^−^ and H^+^, while Cu^2+^ formed microcells with Fe atoms on the steel surface, promoting the oxidation of Fe^2+^ to Fe^3+^ and generating dense oxide films [45] (e.g., FeOOH) to inhibit anodic dissolution [18,19]. However, the MOF particles may agglomerate due to van der Waals forces at 80 mg/L [19], resulting in a decrease in the effective adsorption area and a decrease in corrosion inhibition efficiency.

As for the Schiff–CS@Cu group, the corrosion inhibition rate increased approximately monotonically with the increase in concentration, and *η* peaked at 300 mg/L, indicating a more stable corrosion inhibition performance. It was attributed to the fact that the pyridine ring in the chitosan Schiff bases chelated with Cu^2+^ to form a stable ligand layer, which was chemically bonded to Fe atoms on the steel surface via the N and O heteroatoms [21]. It constructed a bilayer barrier to block the penetration of corrosive media [22]. Its corrosion inhibition efficiency increased linearly with concentration, indicating that the coordination film coverage was positively correlated with the corrosion inhibition performance.

It should be noted that once the concentration of the two inhibitors exceeded 100 mg/L, the growth rate of their corrosion inhibition efficiencies slowed down, which may imply the approaching of their respective critical effects of corrosion inhibition.

### 3.2. Corrosion Morphology Analysis

#### 3.2.1. The Morphology Under the Same Corrosion Inhibitors Concentration

**Figure 3:**
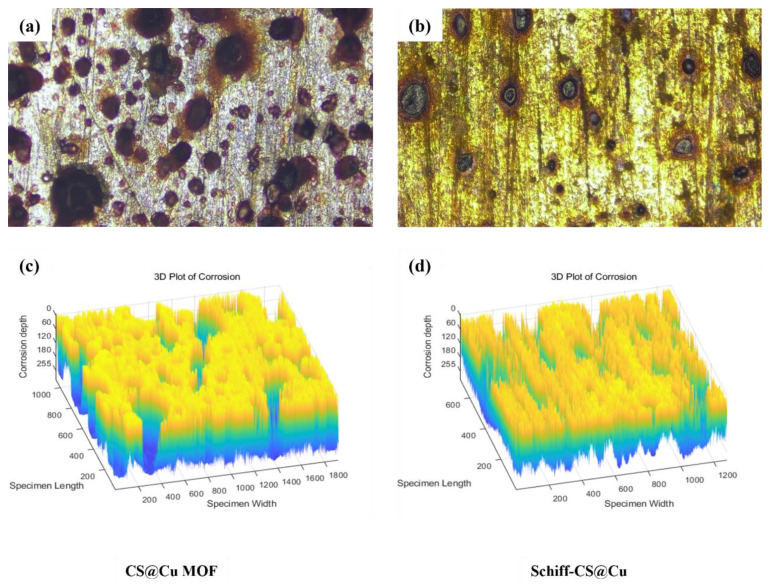
Micro-corrosion morphology of Q345 steel specimens with corrosion inhibitor concentration of 80 mg/L at 20× magnification: (**a**,**c**) CS@Cu MOF; (**b**,**d**) Schiff–CS@Cu MOF.

Figure 3 present the corrosion morphologies of Q345 steel specimens with an inhibitor concentration of 80 mg/L. The distinct differences in surface degradation between CS@Cu MOF and Schiff–CS@Cu groups highlighted their contrasting inhibition mechanisms.

As shown in Figure 3a,c, the CS@Cu MOF-treated specimen exhibited widespread pitting corrosion with irregularly distributed pits at 20× magnification, which indicated insufficient barrier protection. This aligned with the elevated corrosion rate (*v* = 3.167 g/(m^2^·h)) observed at this concentration. Meanwhile, Figure 3b,d presents the Schiff–CS@Cu group shallow pits and sparse corrosion products at 80 mg/L, which explained the lower corrosion rate (*v* = 1.833 g/(m^2^·h)) despite there being a suboptimal concentration.

#### 3.2.2. The Morphology at Lower Corrosion Inhibition Effect

**Figure 4:**
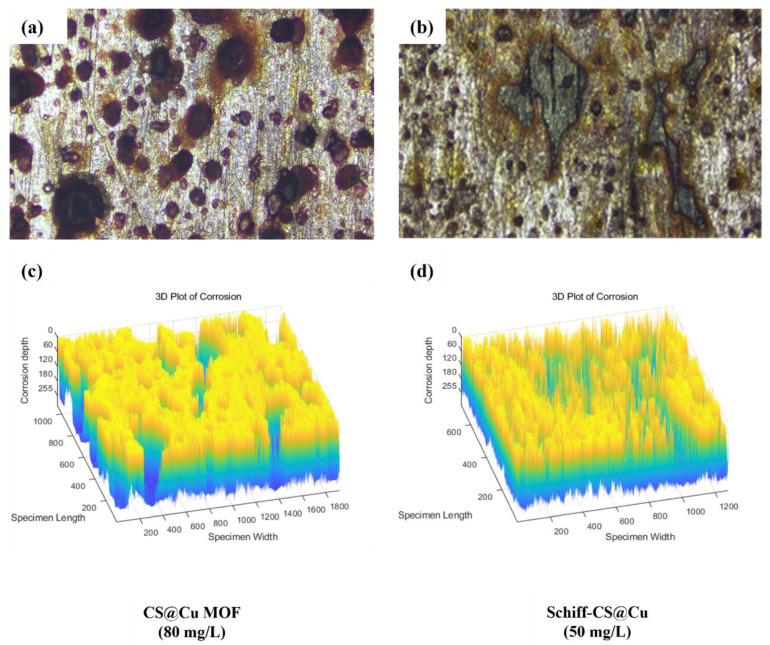
Micro-corrosion morphology of Q345 steel specimens with lower corrosion inhibition effect at 20× magnification: (**a**,**c**) CS@Cu MOF; (**b**,**d**) Schiff–CS@Cu MOF.

The corrosion morphology of Q345 steel samples under the worst corrosion inhibition effect of the two corrosion inhibitors was presented in Figure 4. In this case, the concentrations of CS@Cu MOF and Shichill-CS@Cu were 80 mg/L and 50 mg/L, respectively, which originated from the test results in Figure 2.

From Figure 4a,c, it can be seen that the black corrosion spots on the surface of the steel samples at a CS@Cu MOF concentration of 80 mg/L were denser and larger in area, and accompanied by deeper corrosion pits. However, Figure 4b,d confirms that the number and area of black spots on the sample surface at Schiff–CS@Cu concentration of 50 mg/L were smaller, and the depth of corrosion was shallower (except for a few lumpy and shallow corrosion pits) compared to CS@Cu MOF. This phenomenon indicated that the corrosion inhibition effect of Schiff–CS@Cu on the steel was superior to that of CS@Cu MOF in the respective most unfavorable corrosion inhibition effect, which was consistent with the conclusion of Section 3.1.

The corrosion inhibition performance of CS@Cu MOF and Schiff–CS@Cu composites was evaluated by quantifying corrosion areas at different magnifications (20× and 50×), as presented in Table 2.

The corrosion area of CS@Cu MOF at 80 mg/L was 833,361 and 1,420,465 pixels at 20× and 50×, respectively, which was significantly higher than that of Schiff–CS@Cu at the same concentration of 118,731 and 28,033 pixels. In addition, the corrosion area of Schiff–CS@Cu at 50 mg/L increased to 239,232 and 472,139 pixels. This indicated that the corrosion inhibition effect of Schiff–CS@Cu was superior to that of CS@Cu MOF, especially at high concentrations, and the effect was weakened when the concentration decreased.

The high corrosion area of CS@Cu MOF at 80 mg/L might be due to the aggregation of particles, which resulted in the reduction in the effective surface area and the inability to effectively adsorb corrosive ions. Meanwhile, the porous structure of MOF may adsorb the electrolytes and promote localized corrosion instead [18]. The microcell effect (coupling of Cu^2+^ to Fe) mentioned in the literature may have exacerbated the localized corrosion [19].

The excellent performance of Schiff–CS@Cu at 80 mg/L may have been due to the formation of a dense chemisorption film between Schiff base and Cu^2+^, which effectively blocked the corrosive medium. Conversely, the membrane coverage was incomplete when the concentration was reduced to 50 mg/L, resulting in an increase in the corrosion area [20,21,22].

The above analysis revealed that CS@Cu MOF mainly relied on physical adsorption, while Schiff–CS@Cu formed a protective film through chemical coordination, and the latter was more stable and effective in protecting steel.

## 4. Conclusions

This study focused on the corrosion inhibition performance of chitosan MOF (CS@Cu MOF) and chitosan Schiff base (Schiff–CS@Cu) on Q345 steel in corrosive solution. The corrosion inhibition effect of the two corrosion inhibitors was comparatively analyzed through the design of different concentrations, and the following main conclusions were obtained:

(1) Chitosan MOF (CS@Cu MOF) and chitosan Schiff base (Schiff–CS@Cu) have a favorable corrosion inhibition effect on Q345 steel; the concentration of 100 mg/L is recommended, considering its efficiency and economy.
(2) Schiff–CS@Cu has better corrosion inhibition performance than physical adsorption-dominated CS@Cu MOF due to the chemical ligand-dominated dense film layer.

The dispersibility of MOF needs to be improved by surface functionalization or the development of a MOF-Schiff base composite system to synergistically enhance the corrosion inhibition performance.

## Figures and Tables

**Table 1:** Corrosion rates of CS@Cu MOF and Schiff–CS@Cu groups based on mass loss method.

Items	Codes	Mass Before Corrosion, *m*_1_ (g)	Mass After Corrosion, *m*_2_ (g)	Corrosion Rate, *v* (g/(m^2^·h))
Blank control group	B-0	11.996	11.841	25.889
CS@Cu MOF group	M-30	12.351	12.336	2.500
	M-50	12.446	12.430	2.667
	M-80	13.371	12.352	3.167
	M-100	12.438	12.424	2.500
	M-200	12.435	12.421	2.333
	M-300	12.369	12.357	2.000
Schiff–CS@Cu group	X-30	12.392	12.378	2.333
	X-50	12.345	12.328	2.833
	X-80	12.372	12.361	1.833
	X-100	12.286	12.276	1.667
	X-200	12.226	12.217	1.500
	X-300	12.347	12.339	1.333

**Table 2:** Corrosion area of the samples under the effect of different corrosion inhibitors.

Corrosion Inhibitors	Concentration (mg/L)	Corrosion Area (Pixels)	
		Magnification 20×	Magnification 50×
CS@Cu MOF	80	833,361	1,420,465
Schiff–CS@Cu	80	118,731	28,033
	50	239,232	472,139

## Data Availability

The original contributions presented in this study are included in the article. Further inquiries can be directed to the corresponding authors.
